# Genome-Wide Association Study on Seminal and Nodal Roots of Wheat Under Different Growth Environments

**DOI:** 10.3389/fpls.2020.602399

**Published:** 2021-01-11

**Authors:** Fengdan Xu, Shulin Chen, Xiwen Yang, Sumei Zhou, Xu Chen, Jie Li, Kehui Zhan, Dexian He

**Affiliations:** ^1^College of Agronomy of Henan Agricultural University/National Engineering Research Center for Wheat/Co-construction State Key Laboratory of Wheat and Maize Crop Science/Collaborative Innovation Center of Henan Grain Crops, Henan Agricultural University, Zhengzhou, China; ^2^College of Agronomy, Xinyang Agriculture and Forestry University, Xinyang, China

**Keywords:** GWAS, grain yield, seminal root, wheat, nodal root

## Abstract

The root of wheat consists of seminal and nodal roots. Comparatively speaking, fewer studies have been carried out on the nodal root system because of its disappearance at the early seedling stage under indoor environments. In this study, 196 accessions from the Huanghuai Wheat Region (HWR) were used to identify the characteristics of seminal and nodal root traits under different growth environments, including indoor hydroponic culture (IHC), outdoor hydroponic culture (OHC), and outdoor pot culture (OPC), for three growing seasons. The results indicated that the variation range of root traits in pot environment was larger than that in hydroponic environment, and canonical coefficients were the greatest between OHC and OPC (0.86) than those in other two groups, namely, IHC vs. OPC (0.48) and IHC vs. OHC (0.46). Most root traits were negatively correlated with spikes per area (SPA), grains per spike (GPS), and grain yield (GY), while all the seminal root traits were positively correlated with thousand-kernel weight (TKW). Genome-wide association study (GWAS) was carried out on root traits by using a wheat 660K SNP array. A total of 35 quantitative trait loci (QTLs)/chromosomal segments associated with root traits were identified under OPC and OHC. In detail, 11 and 24 QTLs were significantly associated with seminal root and nodal root traits, respectively. Moreover, 13 QTLs for number of nodal roots per plant (NRP) containing 14 stable SNPs, were distributed on chromosomes 1B, 2B, 3A, 4B, 5D, 6D, 7A, 7B, and Un. Based on LD and bioinformatics analysis, these QTLs may contain 17 genes closely related to NRP. Among them, *TraesCS2B02G552500* and *TraesCS7A02G428300* were highly expressed in root tissues. Moreover, the frequencies of favorable alleles of these 14 SNPs were confirmed to be less than 70% in the natural population, suggesting that the utilization of these superior genes in wheat root is still improving.

## Introduction

Wheat is one of the most widely cultivated food crops all over the world, with approximately one quarter of the global agricultural area dedicated to wheat cultivation, and it is a main source of food for 30% of the people in the world (Liu et al., 2017). With the increasing population, continuous improvement of wheat yield is of great significance for ensuring food security. In addition to photosynthesis, the accumulation of dry mass aboveground is mostly supported by the absorption and transport of water and minerals by root systems. The amount of carbohydrates needed for grain filling can be guaranteed when the aboveground parts have a higher biomass (Gaju et al., 2014; Rogers and Benfey, 2015). Additionally, improving the root architecture in the soil promises to reduce the overprovision of nitrogen fertilizers, impacting the environment and economy and being a major part of the breakthrough for the second “Green Revolution” (Lynch, 2007; Ehdaie et al., 2012).

The narrower is the seminal root angle, the longer is the seminal root length, thereby allowing the roots to easily access residual moisture in deep soils and increase the drought tolerance of wheat (Gao et al., 2016; Hodgkinson et al., 2017). Kirkegaard and Lilley (2007) showed that a 30-cm increase in root depth into the subsoil could capture an extra 10 mm of rainfall water during the critical grain filling stage, increasing the yield by 500 kg • ha^−1^. Because roots are hidden in soil, it is non-ideal and labor-intensive to extract complete roots. Previous studies have succeeded in utilizing indoor cultivation approaches to dissect field root traits in the early root development of wheat, such as using germination paper-based methods (Bai et al., 2013), hydroponic culture (Ren et al., 2012), pots (Cao et al., 2014), paper culture (Atkinson et al., 2015), gel-filled chambers (Christopher et al., 2013), and wax-layer screens (Bai et al., 2019). However, in rice, the ability of roots to penetrate strong wax layers under indoor cultivation showed limited association with accessions that present deep root systems in the field (Clark et al., 2002). Bai et al. (2019) also found that genotypic variation in root depth had a poor correlation between field and laboratory tests and across 2 years. Moreover, the density of roots decreases exponentially with depth in the field (Fan et al., 2016), a finding that is inconsistent with many laboratory results (Jin et al., 2015; Gao et al., 2016). These results showed that it was difficult to predict root growth in the field based on laboratory experiments.

The genetic basis of root development have been mainly clarified in QTL mapping within special populations by using genetic maps in previous research. Ren et al. (2012) detected 35 QTLs for the root length and root tip number of seminal roots by utilizing a recombinant inbred wheat line population of Xiaoyan 54 × Beijing 411, which were distributed on chromosomes 2B, 2D, 4B, 6A, 6B, and 7B. Bai et al. (2013) detected nine QTLs for root length from double haploid lines derived from Avalon × Cadenza on chromosomes 3A, 3B, 4D, 5B, and 6A, explaining a maximum of 16.03% of the phenotypic variations. With the release of the reference genome sequence, many types of high-density SNP arrays designed based on functional genes have been successfully developed to identify significantly associated SNPs and even candidate genes for various traits. Li et al. (2019) identified 93 SNPs for root length and root dry weight by GWAS, and three of them (*Co-6A*, *Co-6B*, and *Co-6D*) were associated with root depth at both booting and mid-grain filling stages. Meanwhile, some vital genes cloned by reverse approaches were also confirmed to putatively participate in regulating root development. For example, F-box (Hua and Vierstra, 2011), zinc finger proteins (Chang et al., 2016), and ABC transport proteins (Gaedeke et al., 2001) could affect lateral root formation. To date, *RHD3* (Shan et al., 2005) and *PIN* family genes (Hochholdinger et al., 2018) have also been reported to control root development.

Wheat root is a type of fibrous root system, which consists of seminal and nodal roots. Nodal roots are the main components of the root system (Maccaferri et al., 2016) and account for more than 70% of the root system during the middle and late growth stages (Steinemann et al., 2015). However, previous studies have mainly focused on QTL analyses for the total root system or seminal root system, and few studies have examined the genetic basis of the nodal root system. Moreover, root research is very labor intensive, and many environmental factors including geographical location, fertilizer, water supply, and cultivation method could affect root development (Osmont et al., 2007), and indoor research alone is not sufficiently comprehensive to study roots. Only a few accessions have been examined in a field or pot in previous studies, because root research is very labor-intensive. Thus, it is necessary to compare the characteristics of root development with the yield and clarify their genetic mechanisms under different growth environments. We selected 196 wheat accessions from the HWR as a natural population to investigate root traits under the three growth environments. GWAS was then performed based on the wheat 660K SNP array. The study not only provides a theoretical basis to predict wheat root development but also screens elite accessions to support root improvement breeding.

## Materials and Methods

### Plant Materials

The 196 selected wheat accessions from the HWR in China, including Henan, Hebei, Shanxi, Shandong, Jiangsu, Shaanxi, Sichuan, Anhui provinces, and Beijing. The source and genetic background of these accessions were consistent with those reported by Chen et al. (2020) (Supplementary Table S1). Seeds were provided by the Collaborative Innovation Center of Henan Grain Crops, Henan Agricultural University.

### Experimental Design

The root used for experiments were planted in one indoor hydroponic environment, three outdoor hydroponic environments, and three outdoor pot environments, and the study was conducted at Zhengzhou (34.7°N, 113.6°E), Henan, China.

#### Hydroponic Culture Experiments

In the hydroponic experiments, seeds of each accession were surface sterilized by soaking in 10% H_2_O_2_ for 10 min, followed by washing with fresh water three times. Next, seeds were grown on germination substrate for 6 days treating with sterile water. Finally, seedlings with robust growth were transferred to plastic boxes (45 cm long, 30 cm wide, and 15 cm high) containing 18 L of nutrient solution. The nutrient solution was operated according to the method described by Ren et al. (2012) with minor modifications. The nutrient solution was refreshed every 3 days, with the pH maintained at 6.0. One hundred seedlings were planted in one box with two seedlings for each accession.

Indoor hydroponic culture (IHC) experiments were performed in a greenhouse with three replicates, and the greenhouse condition was as follows: 16 h/8 h light/darkness with only upper light-source, photoperiod at 20°C/16°C and a light intensity of 180–200 μmol • m^−2^ • s^−1^. Outdoor hydroponic culture experiments were performed from October 20 to November 20 in 2016, 2017, and 2018. A full box of nutrient solution was supplied daily and a movable shelter was prepared to escape rain. All wheat accessions were planted in a randomized block design with three replicates, two plants per replicate in each hydroponic experiment.

#### Pot Experiments

The experimental pots were placed in the field from October 20 to November 20 in 2017, 2018, and 2019 growing seasons. The 196 wheat accessions were planted in a completely randomized design with two replicates in 2017 and 2018, and three replicates in 2019. Each pot (height, 10 cm and diameter, 12 cm) was filled with 3 kg of tillage soil, and the edge of each pot was on the same plane along the ground. The tillage soil was described as “white-sand loam” with a classification of sandy soil, containing fertilizer with nitrogenous 1.02 g • kg^−1^, phosphorus 38.42 mg • kg^−1^, and potassium 134.44 mg • kg^−1^. Before planting, seeds were germinated as describing in hydroponic culture experiments. Uniformly germinated seeds of each accession were planted into pots. Each pot kept two seedlings at 10 days after sowing. To facilitate management, 396/588 pots representing all the accessions were arranged within 20 blocks (length, 150 cm and width, 50 cm), containing 40/60 pots for each block. Borders around the quadrats were set to protect the plants from margin effects. The pots were irrigated at 10-day intervals after sowing, and 70–80% moisture content was maintained. Other field managements were followed local agronomic practices.

Thirty days after sowing, the roots were rinsed with sterile deionized water before measuring root traits. First, every plant was manually investigated for number of seminal roots per plant (SRP) and nodal roots per plant (NRP). Next, a Win-RHIZO system (LA6400XL, Regent Instruments Inc., Quebec, Canada) was used to scan and analyze the root morphology, including average seminal root diameter (ASD), total length of seminal roots (SRL), total seminal root tips (SRT), average nodal root diameter (AND), total length of nodal roots (NRL), and total nodal root tips (NRT). Finally, the roots were oven-dried at 80°C for 48 h to determine dry weight of seminal roots (SDW) and dry weight of nodal roots (NDW) using an analytical balance (Germany SARTORIUS, QUINTIX224-ICN).

#### Field Experiments

All the accessions were planted in four environments at three locations: Zhengzhou (34.7°N, 113.7°E) during 2014–2015 and 2015–2016, Shangqiu (33.4°N, 115.4°E) during 2014–2015, and Zhumadian (33.0°N, 114.1°E) during 2014–2015, with north-south direction planting and two replicates. Each accession contained four rows (each 1.5 m in length), with 23-cm spacing between two rows, and 110 seeds were uniformly sown per row. All the wheat accessions were grown in each block and the management was followed local agronomic practices. Each accession was harvested from the middle rows to calculate the grain yield (GY) and spikes per unit area (SPA). Twenty adjacent spikes were randomly selected to measure grains per spike (GPS) and thousand-kernel weight (TKW).

### Statistical Analysis

The joint variance, descriptive statistics, broad-sense heritability, and best linear unbiased estimate (BLUE) were examined for phenotypic values obtained for all environments in IciMapping v4.0 software (Valassi and Chierici, 2014; Lei et al., 2015), whereas canonical and Pearson correlation coefficients were calculated by using SAS v9.4.

The mean values obtained from each growth environment were used to perform differential analyses, multiple stepwise regression, comprehensive evaluation of the root system, and *t*-test in Excel 2010. For differential analyses, differences in the root traits from different growth environments could be calculated by comparing three test groups between IHC and OHC, OHC and OPC, IHC and OPC. As for the multiple regressions, the predicted yield per hectare was set as the dependent variable (*y*), whereas the root traits under OPC were set as the independent variables (*x*). A comprehensive evaluation of the seminal and nodal roots under different growth environments was conducted according to the standardized normal distribution method (Chen et al., 2020). The equation $u=\left(x-\overset{-}{x}\;\right)/s$, where, for each root trait, 𝑥 is the average value from different years in each growth environment, and *s* and $\overset{-}{x}$ are the standard deviation and arithmetic mean of the 196 accessions, respectively. Then, the average *u* value was calculated for each trait obtained from each growth environment.

### GWAS and Prediction of Candidate Genes

A mixed linear model correcting for both the Q-matrix and K-matrix [MLM (Q + K)] was used to analyze associations among single environment and BLUE values for each trait in Tassel v5.0. Here, the threshold of -log_10_^(*P*)^ was determined at a uniform suggestive genome-wide significance threshold (−log_10_^(*P*)^ ≥ 3.5; Beyer et al., 2019). Manhattan and Q-Q plots were generated using the CMplot package in R.^1^

Alleles with positive effects leading to higher values of grain yield were described as “Superior alleles,” whereas those with lower values were “Inferior alleles.” Superior allele variations of the natural population were then assessed. Haploview 4.2 software was used to analyze the local linkage disequilibrium (LD) and haploblock structure. Identification and expression pattern analyses of the candidate genes within the blocks were performed according to Zheng et al. (2019).

## Results

### Phenotypic Variation in Root Traits Under Different Growth Environments

The frequency distribution for root traits was continuous, and most of them showed a normal or an approximately normal distribution under different growth environments (Supplementary Figure S1). Based on the joint variance analysis, genotypes, and genotypes × environment (G × E) were both significant at *p* ≤ 0.001. The root morphology was different under the three growth environments (Table 1 and Supplementary Table S2). In detail, SRL under OHC (719.63 cm) was much higher than that under IHC (453.68 cm) and OPC (461.76 cm), and the difference in SRL between OHC and IHC and between OHC and OPC were very significant. SRT under OPC (537.86) was slightly higher than that under OHC (508.34), and those under both OPC and OHC were significantly higher than that under IHC (412.47). ASD and SDW under OPC (0.47 mm, 56.10 mg) were the largest, followed by OHC (0.37 mm, 28.39 mg) and IHC (0.34 mm, 14.97 mg). The differences in ASD and SDW among the different growth environments were very significant. SRP was approximately consistent under the three growth environments. The mean coefficients of variation (CVs) of the seminal root system were 20.70% (IHC), 12.94% (OHC), and 20.67% (OPC). The differences in nodal root traits among different growth environments were highly significant. NRL and NRP under OHC (79.82 cm, 4.72) were slightly higher than those under IHC (67.24 cm, 4.21), and those under both OHC and IHC were significantly higher than those under OPC (36.38 cm, 2.21). NRT under OHC (74.42) was the largest, followed by IHC (58.92) and OPC (47.61). AND and NDW under OPC (0.64 mm, 13.76 mg) were the largest, followed by OHC (0.60 mm, 28.39 mg) and IHC (0.45 mm, 6.69 mg). The mean CVs of the nodal root system were 30.63% (IHC), 26.28% (OHC), and 36.79% (OPC). This result indicated that the variation range of root traits in the pot environment was larger than that in the hydroponic environment and the variation ranges of the nodal roots were larger than that of the seminal roots.

**Table 1:** Phenotype variation of the root traits for 196 accessions.

	Traits	IHC				OHC				OPC			
		Range	Mean	CV (%)	*H_B_*^2^	Range	Mean	CV (%)	*H_B_*^2^	Range	Mean	CV (%)	*H_B_*^2^
Seminal root	SRL(cm)	281.15–686.8	453.68	21.93	-	423.29–1032.52	719.63	15.86	0.62	212.31–854.02	461.76	24.31	0.47
	ASD(mm)	0.29–0.43	0.34	8.80	-	0.32–0.42	0.37	5.07	0.50	0.36–0.51	0.44	6.54	0.42
	SRT(count)	208.75–734.25	412.47	31.31	-	271.33–809.67	508.34	18.53	0.56	184.00–1091.00	537.86	39.84	0.48
	SRP(count)	3.33–8.00	5.61	16.36	-	4.17–7.08	5.61	9.05	0.42	3.99–6.68	5.35	9.36	0.34
	SDW(mg)	7.48–24.71	14.97	25.09	-	18.20–39.60	28.39	15.55	0.50	29.30–99.40	56.10	23.73	0.39
Nodal Root	NRL(cm)	11.33–169.69	67.24	34.6	-	37.36–138.24	79.72	28.14	0.56	12.65–88.02	36.38	43.10	0.38
	AND(mm)	0.30–0.60	0.45	14.95	-	0.41–0.74	0.60	9.19	0.53	0.49–0.79	0.64	10.79	0.42
	NRT(count)	14.13–127.50	58.92	36.75	-	23.76–150.42	74.42	39.00	0.55	12.3–122.75	47.61	52.02	0.36
	NRP(count)	1.25–8.65	4.21	32.91	-	2.83–7.60	4.72	16.40	0.53	1.37–3.50	2.21	16.97	0.44
	NDW(mg)	1.00–14.90	6.69	33.94	-	3.50–13.80	8.48	27.78	0.54	4.80–31.90	13.76	37.3	0.38

SRL, total length of seminal roots; ASD, average seminal root diameter; SRT, total seminal root tips; SDW, dry weight of seminal roots; SRP, seminal roots per plant; NRL, total length of nodal roots; AND, average nodal root diameter; NRT, total nodal root tips; NDW, dry weight of nodal roots; NRP, nodal roots per plant; OHC, outdoor hydroponic culture; IHC, indoor hydroponic culture; OPC, outdoor pot culture; CV, coefficient of variation; H_B_^2^, broad sense heritability; −, indicated not hereditary component. The bellows are the same.

**Figure 1:**
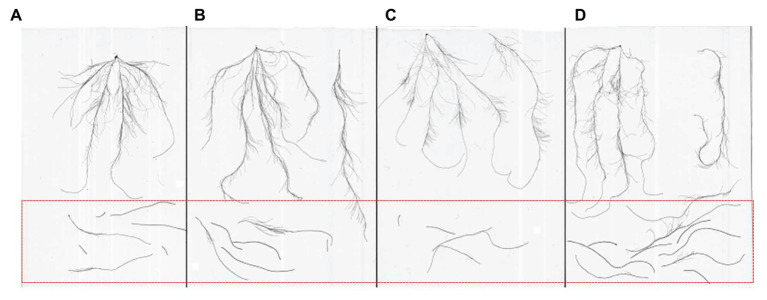
Different types of roots **(A)** accessions with small seminal root; **(B)** accessions with large seminal root; **(C)** accessions with small nodal root; and **(D)** accessions with large nodal root; roots within the red frame are nodal roots, and unexpected seminal roots.

Additionally, based on standardized normal distribution (Supplementary Table S1), some accessions had approximate *u* values under the three growth environments. For instance, the seminal root system development of Xinmai 18, Pu 2056, and Kaimai 21 was worse with lower *u* values, while Xinong 529, Nanda 2,419, Zhengpinmai 8, and Luomai 28 developed roots quickly, as demonstrated by their higher *u* values. Nodal root system development of Pingan 8, Zhongyu 12, Zhongmai 1, and Dan 6172 cultivars was worse with lower *u* values, while Chinese Spring and Xinong 529 developed roots quickly, as demonstrated by their higher *u* values (Figure 1).

For IHC, the SRL was the longest in Yujiao 5 and Liangxing 99 and the shortest in Yumai 68 and Haocheng 8901. The SRT was the highest in Jinan 17 and Shangmai 156 and the lowest in Hengguan 35 and Zhoumai 18. The SRP was the highest in Luohan 6 and the lowest in Fanmai 803. The SDW was the largest in Zongmai 1 and Huaichuan 919 and the smallest in Hengguan 35. Xinmai 19 and Yunong 416 had longer NRLs and higher NRTs. Liangxin 99 and Qiule 2122 had lower NRPs, shorter NRLs and lower SRTs, and Zhengmai 583 had a higher NRP. The NDW was the largest in Yumai 49 and Lunxuan 1298 and the smallest in Zhengmai 004.

### Correlations of Root Traits Under Different Growing Environments

Canonical correlation analysis showed that the strongest correlation of root traits among the three growth environments was between OHC and OPC (0.86), followed by OHC vs. IHC (0.49) and then IHC vs. OPC (0.46). Except for NRT and NDW, the correlation coefficients of all root traits were significant between OHC and OPC. Between IHC and OHC, the correlations of seminal roots were approximately 0.1, and the correlations of nodal roots were low or negative. However, the correlations between IHC and OPC were low, with correlation coefficients close to zero (Table 2). The above results showed that the root morphology under IHC and OPC was different, indicating that the consistency of root development between IHC and the field was low. Therefore, the root phenotype under IHC was not further analyzed. The correlations among the roots between OHC and OPC were the highest, thus, we focused on analyzing root traits under OHC and OPC in the next step.

**Table 2:** Phenotypic associations among OHC, IHC, and OPC.

	Traits	OHC-VS-OPC	IHC-VS-OHC	IHC-VS-OPC
Seminal root	SRL	0.29^**^	0.10	0.10
	ASD	0.16^*^	0.11	0.16^*^
	SRT	0.32^**^	0.15^*^	0.19^**^
	SRP	0.31^**^	0.19^*^	0.23^**^
	SDW	0.32^**^	0.07	0.14
Nodal root	NRL	0.19^**^	0.08	0.02
	AND	0.19^**^	0.16^*^	0.13
	NRT	0.10	0.15	−0.01
	NRP	0.26^**^	0.11	−0.10
	NDW	0.07	0.01	−0.02
	*V*	0.86^**^	0.49^**^	0.46^**^

VS indicates versus, V represents the canonical correlation coefficient.

** Represents that an extremely significant level *p* < 0.01

* Represents the significant level *p* < 0.05.

### Correlations of Root Traits With Yield Traits

Most root traits in both OHC and OPC were negatively correlated with GY, SPA, and GPS. In detail, SPA was significantly correlated with AND (−0.26) under OPC and NDW (−0.26) under OHC (*p* < 0.01). GY was significantly correlated with SRL (−0.16), SRT (−0.26), and NRT (−0.15) under OPC, and SRL (−0.158), SRT (−0.257) and four nodal root traits under OHC (*p* < 0.05). Five seminal root traits in both OHC and OPC were positively correlated with TKW while nodal root traits showed a weak correlation with TKW (Supplementary Table S3). These results showed that root traits were negatively correlated with GY, especially nodal root traits.

Multiple stepwise regression analysis found that yield (*y*) was significantly correlated with SRL(x_1_), SRT (x_2_), SDW (x_3_), and NRP (x_4_) under OPC. The best-fitting regression equation was *y* = 694.39–0.21x_1_–0.30x_2_–0.91x_3_–33.81x_4_. Larger SRL, SRT, SDW, and NRP values did not increase yield, and the partial regression coefficient of NRP was larger than that of SRL, SRT, and SDW (Table 3).

**Table 3:** Stepwise regression of the yield (y) with Root traits (x) under OPC.

Trait	*p*	Stepwise regression	*R*^2^
SRL(x_1_)	4.24E-03	y = 694.39–0.21x_1_–0.30x_2_–0.91x_3_–33.81x_4_	0.22
SRT(x_2_)	8.79E-05		
SDW(x_3_)	1.12E-02		
NRP(x_4_)	9.25E-04		

Upon analyzing the root characteristics of the accessions with the 20 highest and 20 lowest yields, it was found that the range of root variation in low-yield accessions was larger than that in high-yield accessions (Table 4). Compared with the mean values of all accessions, the low-yield accessions belonged to a maximum or minimum root system, high-yield accessions presented a medium or even slightly smaller root system. Moreover, the differences in SRT, SDW, NRL, and NRT were significant between the high-yield and low-yield accessions.

**Table 4:** Differences of the root traits between 20 lower and 20 higher yield accessions under OPC.

	Traits	High yield			Low yield				
		Mean	Range	CV (%)	Mean	Range	CV (%)	D-value	*p*
Seminal	SRL	432.18	328.94–576.99	18.16	503.25	307.66–774.15	28.78	−71.07	0.05
root	ASD	0.44	0.41–0.50	5.74	0.44	0.37–0.51	7.40	0.002	0.38
	SRT	515.25	390.69–782.97	19.17	622.08	293.28–1070.67	39.53	−106.83	0.01
	SRP	5.18	4.14–6.18	8.84	5.33	4.00–6.51	11.48	−0.15	0.20
	SDW	52.69	35.30–71.10	20.08	64.25	32.50–95.60	25.50	−11.57	0.01
Nodal	NRL	29.35	13.55–45.13	33.96	39.16	15.63–70.66	42.62	−9.81	0.01
root	AND	0.64	0.51–0.77	10.19	0.64	0.51–0.79	11.80	0.000	0.50
	NRT	37.64	15.55–62.77	34.46	52.4	12.95–116.63	53.19	−14.76	0.03
	NRP	2.18	1.37–3.01	18.35	2.37	1.58–3.48	21.49	−0.19	0.11
	NDW	13.91	7.60–31.90	38.25	14.31	5.40–22.30	34.99	−0.40	0.41

D-value indicated differences in root traits over 2 years under OPC, the difference value was calculated using low and high yield accessions; value of *p* for each trait was based on t-test comparisons of data from low and high yield accessions.

### GWAS of Wheat Roots

By a quality control check of the genotypic data, the wheat 660K SNP array identified 390,136 polymorphic SNPs with MAF > 5%. Three chromosome groups were detected, with 148,386 (A), 188,464 (B) and 45,995 (D) SNPs. GWAS was performed between the SNPs and root traits of each environment and the BLUE values were determined the optimal of mixed linear model. In three OHC environments, a total of 1,404 SNPs were detected, with an *R*^2^ ranging from 6.50 to 18.01%. In three OPC environments, a total of 1,288 SNPs were detected, with an *R*^2^ ranging from 6.97 to 15.70% (Supplementary Table S4). The heritabilities of root traits were low (*H_B_*^2^ <60%), and the significant SNPs based on BLUE values detected under OHC and OPC were used for further analysis (Table 5 and Supplementary Table S5).

**Table 5:** Summary of significant SNPs by GWAS.

	Traits	OHC				OPC				Share
		SNP	QTL	-log_10_^*(p*)^ Range	R^2^ (%) Range	SNP	QTL	-log_10_*^(p)^* Range	R^2^ (%) Range	QTL
Seminal root	SRL	49	30	3.53–4.98	7.10–15.00	45	18	3.53–4.44	7.03–12.05	1
	ASD	75	14	3.75–5.19	7.56–11.46	15	9	3.54–4.26	7.14–10.14	0
	SRT	63	27	3.50–5.00	7.13–13.98	150	34	3.52–8.16	7.24–21.40	6
	SRP	13	12	3.51–4.33	7.15–10.83	6	5	3.56–3.77	7.06–10.00	2
	SDW	44	16	3.51–4.33	7.15–10.83	14	12	3.53–4.36	8.87–10.94	2
	Seminal root total	244	99			230	78			11
Nodal Root	NRL	78	49	3.53–5.70	7.16–18.44	80	18	3.53–4.90	7.06–12.35	6
	AND	36	18	3.56–4.83	7.13–12.47	60	15	3.52–4.49	7.15–13.25	1
	NRT	124	66	3.53–6.18	7.07–16.33	61	14	3.52–5.93	7.21–14.77	1
	NRP	69	28	3.51–4.73	9.04–22.95	62	26	3.50–4.99	7.05–14.61	13
	NDW	52	27	3.53–4.98	7.10–15.00	63	27	3.53–4.62	7.04–11.87	3
	Nodal Root total	359	188			326	100			24
Total		603	287			556	178			35

SNP, single-nucleotide polymorphisms and QTL, quantitative trait loci.

#### Seminal Root

Under OHC and OPC, 224 and 230 significant SNPs were detected for the seminal traits, respectively, while the *R*^2^ values, which are equivalent to the phenotypic variation explained by SNPs, ranged from 7.03 to 21.40%. The LD decay distance (10 Mb) was set as a confidence interval for a QTL, and thus 99 and 78 QTLs were identified from the significant SNPs (Table 5). Seminal root traits were associated with 11 QTLs in both OHC and OPC, which were distributed on chromosomes 1A, 1B, 1D, 2A, 2B, 3B, 4B, 5A, and 5B, with one QTL for SRL, six QTLs for SRT, two QTLs for SRP, and two QTLs for SDW (Table 6).

**Table 6:** List of 35 stable QTLs detected from GWAS panel under both OHC and OPC.

	Traits	Chromosome	Position	OHC		OPC	
				SNP	*R*^2^ (%)	SNP	*R*^2^ (%)
Seminal root	SRL	1D	464.38–465.41	1	7.21	1	9.25
	SRT	1B	13.46–16.30	2	7.29–9.82	1	14.15
		2B	753.95–761.99	2	9.05	14	7.29–14.53
		3B	40.15–41.97	1	13.98	1	9.65
		4B	26.49–27.52	1	7.63	1	11.05
		5A	3.20–7.58	1	7.54	5	8.41–10.03
		5B	687.22–685.04	1	10.45	1	9.88
	SRP	1A	495.73–504.26	1	10.83	1	9.32
		2A	748.54–753.74	1	7.45	1	9.52
	SDW	2B	11.46–12.60	2	9.80–10.46	1	9.85
		6D	472.28–463.98	1	9.70	1	10.47
Nodal root	NRL	1B	105.69–116.94	1	9.04	1	9.71
		1D	485.44–487.28	1	7.76	1	7.18
		5A	18.69–32.19	2	11.26–12.18	2	9.09–9.15
		6A	2.80–3.02	1	14.55	1	10.35
		7A	696.51–693.81	1	7.94	1	11.01
		Ns	29.52–30.85	1	7.43	1	12.35
	AND	1A	29.61–32.56	1	9.87	1	9.45
	NRT	Ns	482.35–490.42	1	9.08	2	10.25–14.08
	NRP	1B	94.83	1	9.54	1	12.22
		2B	748.43	1	8.67	1	7.91
		2B	777.55	1	10.40	1	10.10
		3A	8.33	1	11.77	1	11.64
		4B	545.77	1	9.28	1	8.72
		4B	621.33	1	9.77	1	8.68
		5D	549.9	1	9.39	1	13.43
		6D	3.05	1	11.44	1	11.36
		7A	621.58	1	8.70	1	9.25
		7A	705.28	1	10.05	1	9.06
		7B	328.14	1	11.00	1	8.79
		7B	711.46	2	7.56–7.68	2	8.16–8.22
		Ns	81.78	1	9.27	1	9.09
	NDW	2A	707.06–717.22	1	9.25	1	9.29
		6B	712.48–714.95	1	8.07	1	7.51
		Ns	486.54–487.98	1	10.34	1	8.23

One QTL was associated with SRL within the 464.38–465.41 Mb region on chromosome 1D, with 1 SNP each in OHC and OPC, explaining 7.21% of the phenotypic variation in OHC and 9.25% in OPC. This QTL was approximately 28 Mb apart from *Xbarc62_1D* (493.48 Mb; Maccaferri et al., 2016). Six QTLs for SRT were detected on chromosomes 1B, 2B, 3B, 4B, 5A, and 5B. Among them, within the 753.95–761.99 Mb region on chromosome 2B, 2 and 14 SNPs were significantly associated with SRT under OHC and OPC, explaining 9.05% and 7.29–14.53% of the phenotypic variation. In particular, *AX-111466536* conferred the highest phenotypic variation of seminal traits in the present study. Another SRT related QTL (3.20–7.58 Mb) on chromosome 5A explaining 7.54% of the phenotypic variation in OHC and 8.05–10.03% of that in OPC, was also near to (8.24 Mb) the linked marker *Xwmc51_5A* reported by Xie et al. (2017). Two QTLs for SRP was from chromosome 1A and 2A. In the 495.73–504.26 Mb region of chromosome 1A, 1 SNP was significantly associated with SRP in each OHC and OPC experiment, explaining 9.32% of the phenotypic variation in OHC and 10.08% in OPC. Another QTL for SRP (748.54–753.74 Mb) on chromosome 2A explained 7.45% of the phenotypic variation in OHC and 9.52% in OPC, which was approximately 30 Mb away from the previous reported marker *Xcfd168_2A* (717.94 Mb; Maccaferri et al., 2016). The other two QTLs for SDW were identified from chromosomes 2B and 6D. The QTL identified in the current study (11.46–12.60 Mb) on chromosome 2B that explaining 8.80–11.46% of the phenotypic variation in OHC and 9.85% in OPC, was 13.40 Mb far away from the linked marker *wpt-8737_2B* according to the physical position. Within the 463.98–472.28 Mb region on chromosome 6D, 1 SNP was detected in each OHC and OPC, explaining 9.70% of the phenotypic variation in OHC and 10.47% in OPC (Table 6).

#### Nodal Root

Additionally, 359 and 326 significant SNPs were detected for the nodal traits in the OHC and OPC, with individual SNP *R*^2^ values ranging from 7.06 to 22.95%, respectively, and 188 and 67 QTLs were identified from the significant SNPs, respectively (Table 5). Nodal root traits in both OHC and OPC were associated with 24 QTLs distributed on chromosomes 1B, 1D, 2A, 2B, 3A, 4B, 5A, 5D, 6A, 6D, 7A, and 7B and Un. Among them, six QTLs were for NRL, one QTL for ARD, one QTL for NRT, 13 QTLs for NRP, and three QTLs for NDW. These QTLs for nodal roots have never been observed before indicating they were novel (Table 6).

**Figure 2:**
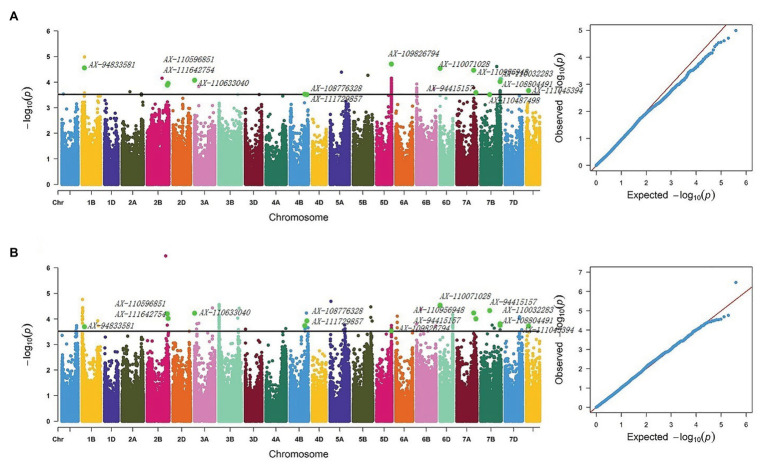
Manhattan and Q-Q plots for NRP **(A)** Manhattan and Q-Q plots based on OPC BLUE values of OPC and **(B)** Manhattan and Q-Q plots based on OHC Blue values of OHC. NRP, nodal roots per plant; BLUE, the best linear unbiased estimate.

Six QTLs for NRL were detected on chromosomes 1B, 1D, 5A, 6A, 7A, and Un, explaining 7.43–14.55% of the phenotypic variation in OHC and 7.18–12.35% in OPC. Among them, within the 2.80–3.02 Mb region on chromosome 6A, 1 SNP was significantly associated with NRL in each OHC and OPC, explaining 10.35% of the phenotypic variation in OHC and 14.55% in OPC. Additionally, *AX-110484513* contributed to the highest phenotypic variation in nodal traits. The QTL with 1 SNP in each OHC and OPC, that was significantly associated with AND on chromosome 1A (3.67–3.96 Mb), explained 9.87 and 9.45% of phenotypic variations in OHC and OPC, respectively. One QTL, from Un chromosome with 1 and 2 SNPs in OHC and OPC, respectively, was significantly associated with NRT (81.78 Mb) and explained 9.08% of the phenotypic variation in OHC and 10.25–14.08% in OPC, respectively. Thirteen QTLs for NRP distributed on chromosomes 1B, 2B, 3A, 4B, 5D, 6D, 7A, and 7B and Un contained 14 repetitive SNPs (*AX-111045394*, *AX-108804491*, *AX-110032283*, *AX-110071028*, *AX-110956948*, *AX-94415157*, *AX-110487498*, *AX-94833581*, *AX-111642754*, *AX-110596851*, *AX-110633040*, *AX-108776328*, *AX-111729857*, and *AX-109826794*), and explained 7.56–11.77% of the phenotypic variation in OHC and 7.91–13.43% in OPC (Figure 2). Three QTLs for NDW were detected in the 707.06–717.22 and 712.48–712.48 Mb region on chromosome 2A and 6B, and in the 486.54–487.98 Mb region on chromosome Un, explained 8.07–10.34% of the phenotypic variation in OHC and 7.51–9.29% in OPC (Table 6).

Under OHC and OPC, 43 and 45 SNPs (Supplementary Table S6) were associated with two or more root morphology traits, respectively. Among them, 13 SNPs were associated with three root morphological traits in OHC and 1 SNP in OPC. For example, *AX-95170701* on chromosome 2D was co-localized with NRL, AND, NRT under OPC.

### Utilization and Distribution of Superior Alleles in the Population

Based on registered time, the natural population used in the present study could be divided into five groups: before 1980s, 1980–1990s, 1990–2000s, 2000–2010s, and after 2010s, and their percentages were 1.53, 3.06, 12.24, 30.61, and 46.94%, respectively. For NRP, 11 of 14 SNPs for the differences in phenotypic values were significant (*p* < 0.05) based on a *t*-test (Supplementary Table S7). Twenty-eight cultivars having more than 10 superior alleles presented averagely nodal roots number of 4.38 (ranging from 3.22 to 5.10) and 2.06 (ranged from 1.48 to 2.53) under OHC and OPC, respectively. Beijing 411 harbor any superior alleles and showed an average nodal root number of 6.56 (OHC) and 3.23(OPC). Further analysis indicated that the total percentages of superior alleles of 14 SNPs were 30.95, 48.81, 56.25, 69.88, and 65.69% for five groups. The frequencies of superior alleles increased over time, although their utilization was still less than 70% in modern cultivars.

### Haploblock Analysis and Prediction of Candidate Gene

**Figure 3:**
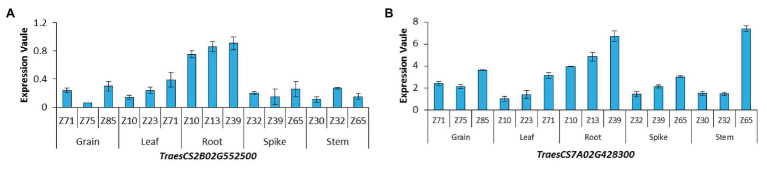
Expression patterns of genes in different wheat organs at three growing stages **(A)** *TraesCS2B02G552500*; **(B)** *TraesCS7A02G428300*; z10, first leaf through coleoptile; z13, three leaves unfolded; z23, main shoot and three tillers; z30, pseudostem erection; z32, second detectable node; z39, flag leaf ligule and collar visible; z65, 1/2 of flowering complete; z71, kernel (caryopsis) watery ripe; z75, medium milk; z85, soft dough.

Haploblock analysis was performed for the 14 stable SNPs. *AX-110596851* and *AX-110956948* on chromosomes 2B and 7A exceeded the experimental threshold of -log_10_^(*p*)^ ˃ 4 and explained from 8.70 to 11.77% of the phenotypic variance, respectively. These two SNPs distributed in the 500-kb regions on both sides were subjected to haplotype analysis. At *r*^2^ = 0.1, one and two blocks were identified in the chromosomal segments on 2B and 7A, respectively. The block on chromosome 2B was located in the region of 748.14–748.51 Mb, which contained four genes. The two blocks on chromosome 7A were respectively located in the region of 621.01–621.58 and 621.80–622.12 Mb, which contained a total of eight genes (Supplementary Figure S2). From the gene annotation (IWGSC RefSeq v1.0), these genes were found to be mainly involved in multiple biological processes, including energy metabolism enzymes, resistance proteins, peroxidase precursors, mitochondrial transcription, and transport. In rice and *Arabidopsis*, 11 and 10 genes were orthologous, respectively (Supplementary Table S8). The expression of 12 genes in different wheat tissues were downloaded (Supplementary Table S9) from the WheatExp website. Among these genes, six were expressed in all the tissues, i.e., root, stem, leaf, spike, and grain, two were only expressed in some special tissues, and the remaining four genes were not expressed. In particular, *TraesCS2B02G552500* and *TraesCS7A02G428300* showed high expression levels in roots at three plant growth stages (Figure 3). These two genes may regulate the occurrence of NRP.

## Discussion

### Impacts of Different Growing Environments on Wheat Root Morphology

Root morphology is the result of the adaptation of plants to the environment, and the spatial configuration of roots changes when plants respond to various biological and abiotic stresses. Previous studies have shown that changes in temperature could significantly affect the biomass and growth of the root system (Alexander et al., 2015). Root morphology was regulated by plant hormone including ethylene production under high temperature environments (Qin et al., 2007). What’s more, light could also induce hormone synthesis and cell differentiation to promote branching of the root system and limit the formation of adventitious roots (Sorin et al., 2005; Molas et al., 2006). In the present study, root morphology also presented abundant phenotypic variation in our GWAS panel under indoor growth environment. A comparison of the root morphology among different growth environments indicated that more dry weight accumulation occurred under outdoor conditions than indoor conditions. The phenomenon may be due to the stronger natural light intensity and larger difference in temperature between day and night outdoors, which will increase photosynthesis but reduces the aerobic respiration of plants. Thereby, this will increase the accumulation of carbohydrates and photosynthesis products for the roots underground. The lower *H_B_*^2^ of root was low for both OHC and OPC, indicating that the root system is indeed a polygenic controlled trait.

Based on the correlations of root traits under different growth environments, we found that the coefficients between OHC and OPC were highly consistent than those between IHC and OPC. For example, Taikong 6 under OPC and OHC had a large seminal root system with a higher *u* value (>1) but a smaller seminal root system with a lower *u* value (0.38) under IHC. By contrast, Xumai 0054 had a smaller nodal root system with a lower *u* value (<−1) but a larger nodal root system with a higher *u* value (0.58) under IHC. However, some accessions had consistent *u* values under the three growth environments showing their strong adaptability to the environment. For instance, Xinmai 18, Pu 2056, and Kaimai 21 had a worse seminal root system with shorter SRL and lower SRT than the other cultivars. Xinong 529, Nanda 2419, Zhengpinmai 8, and Luomai 28 had longer SRL, higher SRT, and larger SDW and belonged to the cultivars with developed seminal root systems. Pingan 8, Zhongyu 12, Zhongmai 1, and Dan 6172 had a worse nodal root system with shorter NRL and lower SDW. Chinese Spring and Xinong 529 had longer NRL, higher NRT values, and larger NDW and thus should belong to the cultivars with developed nodal root systems.

### Relationships Between Root and Yield Traits

The total length of root and seminal root number were positively correlated with the spike number per plant and grain number per spike (Man et al., 2015; Xie et al., 2017; Zheng et al., 2019). In dry years, the root traits are positively correlated with yield of wheat, whereas in other environments, the correlation is negative (Středa et al., 2012). Ma et al. (2008) also demonstrated that reducing the root number can improve water use efficiency in the late flowering stage of wheat, lead to alleviated the competition for resources of plant internal assimilates, and increase the yield under normal conditions. In this study, most root traits were negatively correlated with SPA, GPS, and GY, and all nodal root traits were negatively correlated with yield traits. Moreover, the partial regression coefficients of GY with SRT, SDW, and NRT were negative values, which showed that excess roots were disadvantageous for increasing grain yield. We suspected that roots as a secondary library of photosynthetic products, roots had a competitive relationship with aboveground parts for the growth and development of plants. The root system is indirectly correlated with the yield, and the amount of nutrients absorbed mainly affected the development of the aboveground organs (e.g., leaves) in turn.

The seminal root system can reach 150–200 cm in depth and absorb water and nutrients from deep soil (Osmont et al., 2007). The nodal root system is mainly distributed at 0–40 cm in soil, which is the main functional root system during the late growth stage of wheat (Steinemann et al., 2015). In this study, the accessions with maximum or minimum root types were corresponded to low-yield types, and the root systems of high-yield cultivars had medium or even smaller roots. Overdeveloped roots may cause too much absorption of nutrients, leading to outgrowth at the seedling stage. For overgrown seedlings, the speed of root growth is faster in the early stage, and the better upper root quantity is high, but deep water is not fully used at the later stage of growth. Overgrown of plants could result in uneven absorption of water in each layer of soil, which is an important reason for the decreased yield. When the root system is too small and the amount of total root and root activity are low that the nutrients and water obtained from the soil are insufficient. However, when the development of the aboveground parts is slow, the tillers will be reduced. In addition, the population of wheat in the field is too small, and the number of spikes per unit area is reduced, conditions that are beneficial to yield forming. The root system of strong seedlings grows fast in the later stage of growth, and the amount of deep root is more, allowing full absorption of the deep soil and enhancing the plant resistance ability and improving the yield (Ma et al., 2010; Huang et al., 2012). There is also evidence that the roots of strong seedlings are conducive for uptaking of soil resources (such as N and P) during the early growth stage of plants (Cao et al., 2014). Wheat grows mostly in arid and semiarid areas (Monneveux et al., 2012), rational optimization of root configuration for the formation of strong seedlings not only improves the utilization of deep water and nutrients but also relieves internal competition in plants, thus ensuring a stable and higher yield of wheat.

### Genetic Basis of Wheat Root Traits

Previous studies identified hundreds of QTLs for root traits at the seedling in greenhouses. Soriano and Alvaro (2019) reported a meta-analysis of 754 QTLs in roots and found many consensus regions for root traits in wheat. These QTLs were distributed on all chromosomes, 39% were identified in genome A, 42% in genome B, and 19% in genome D, with an average of 36 QTLs per chromosome, explaining 1.4–76.2% of the phenotypic variance. In this study, a total of 35 QTLs were associated with root traits, and these QTLs were mainly distributed on 1A, 1B, 1D, 2A, 2B, 3A, 4B, 5A, 5B, 6D, 7A, 7B, and Un. Moreover, GWAS identified 14 stable SNPs associated with NRP in OHC and OPC. Functional annotation of them showed that *TraesCS7A02G428200* and *TraesCS7A02G428400* encoding members of the haem peroxidases superfamily, and peroxidase are involved in root duct lignification (Tokunaga et al., 2009) and root hair-specific expression (Li et al., 2018). The cytosine methyltransferase MET1, encoded by the *AT1G18040* gene in *Arabidopsis*, is a protein involved in silencing of the FWA paternal allele in the endosperm (Kim et al., 2014). Two lines with RNAi constructs directed against genes for silencing showed reduced *Agrobacterium*-mediated tumor formation in roots, and the mRNA presented cell-to-cell mobility (Liu et al., 2018). *Os04t0641000* in rice also encodes cytosine methyltransferase (C-5 cytosine-specific DNA methylase; Pound et al., 2013). *TraesCS2B02G552400* is homologous to *AT1G18040* and *Os04t0641000* in hexaploid wheat. However, to date, the function of this gene has not been reported so far. We speculate that this gene may regulate the formation of root cells through cytosine methyltransferase, thus affecting NRP. Expression data for the candidate genes *TraesCS2B02G552500* and *TraesCS7A02G428300* revealed high expression levels in roots at three plant growth stages. *TraesCS7A02G428300* encodes a zinc finger domain protein that potentially plays a vital role in regulating plant growth and development (Chang et al., 2016). Previous studies have found that superior alleles were beneficial to molecular breeding. Yumai 47 and Zhengmai 366 possessed 13 superior alleles, resulting in few NRPs in each environment with average yields of 8670.0 and 8739.6 kg • ha^−1^, respectively. Beijing 841 possessed no superior alleles among the 14-NRP SNPs, that resulting in many NRPs in each environment, and the average yield (7159.8 kg • ha^−1^) was significantly lower than that of Yumai 47 and Zhengmai. However, the utilization of superior alleles remains less than 70% in modern cultivars, thus they should be properly integrated to increase wheat yield.

The GWAS also found that the associated SNPs for one root trait were clustered on different regions of chromosomes or on multiple chromosomes, and some SNPs were co-localized. For example, 13 QTLs were associated with NRT distributed on nine chromosomes. One of them (*AX-110956948* and *AX-109497868*) on chromosome 7A were overlapped with *Xbarc195* (622.1 Mb) for total root dry weight (Maccaferri et al., 2016; Ren et al., 2017). These pleiotropic SNPs might be located in chromosomal regions harboring multi-linked genes or encoding transcription factors. A similar phenomenon was observed in *FLOWERING LOCUS C* (*FLC*), a MADS box gene encoding a transcription factor, that inhibits flowering. *FLC* not only regulates growth habits in spring but also affects flowering, root length, and nitrogen uptake (Lei et al., 2018; Voss-Fels et al., 2018).

## Conclusion

Root correlations were low in OHC vs. IHC and IHC vs. OPC, and all the differences in the nodal root traits among different growth environments were very significant. It is difficult to predict morphology of roots grown in the field based on greenhouse experiments. Most root traits were negatively correlated with SN, GNPS, and GY, while all the seminal root traits were positively correlated with TKW. Neither an oversized nor undersized root system resulted in grain yield increase. However, the high-yield accessions belonged to varieties with a medium or even slightly smaller root system.

A total of 35 QTLs were found to be associated with root traits by GWAS under OPC and OHC, distributed on 18 chromosomes, except for 2D, 4D, and 7D. Moreover, 14 stable SNPs for NRP were detected. Haplotype analysis and annotation revealed 12 candidate genes that encoding proteins involved in various functions. High expression of *TraesCS2B02G552500* and *TraesCS7A02G428300* were found in roots tissues at three growth stages. However, the superior genes of NRP showed a percentage of less than 70% in the GWAS panel, suggesting that there has still been enough space for exploiting these superior genes of in wheat root breeding.

## Data Availability Statement

The original contributions presented in the study are included in the article/Supplementary Material, further inquiries can be directed to the corresponding authors.

## Author Contributions

FX, KZ, and DH conceived the topic. KZ and SC provided gene chip and wheat material. XY and SC helped to draft the manuscript. FX, SZ, XC, and JL performed the phenotypic evaluation and helped with data analysis. FX supervised the whole study and provided assistance for manuscript preparation. All authors contributed to the article and approved the submitted version.

### Conflict of Interest

The authors declare that the research was conducted in the absence of any commercial or financial relationships that could be construed as a potential conflict of interest.

## Footnotes

^1^https://github.com/YinLiLin/R-CMplot

## Abbreviations

SR: Total length of seminal roots
ASD: Average seminal root diameter
SRT: Total seminal root tips
SDW: Dry weight of seminal roots
SRP: Number of seminal roots per plant
NRL: Total length of nodal roots
AND: Average nodal root diameter
NRT: Total nodal root tips
NDW: Dry weight of nodal roots
NRP: Number of nodal roots per plant
GY: Grain yield per area
SPA: Spikes per area
GPS: Grains per spike
TKW: Thousand-kernel weight
OHC: Outdoor hydroponic culture
IHC: Indoor hydroponic culture
OPC: Outdoor pot culture
HWR: Huanghuaihai wheat region
GWAS: Genome-wide association study
BLUE: Best linear unbiased estimation
LD: Linkage disequilibrium
