# Supplementary material for: Genome-Wide Association Study on Seminal and Nodal Roots of Wheat Under Different Growth Environments
Source: Front Plant Sci. 2021 Jan 11;11:602399. doi: 10.3389/fpls.2020.602399 (PMC7829178; doi:10.3389/fpls.2020.602399)
Supplement: Supplementary file 2 [file Data_Sheet_1.pdf]

A

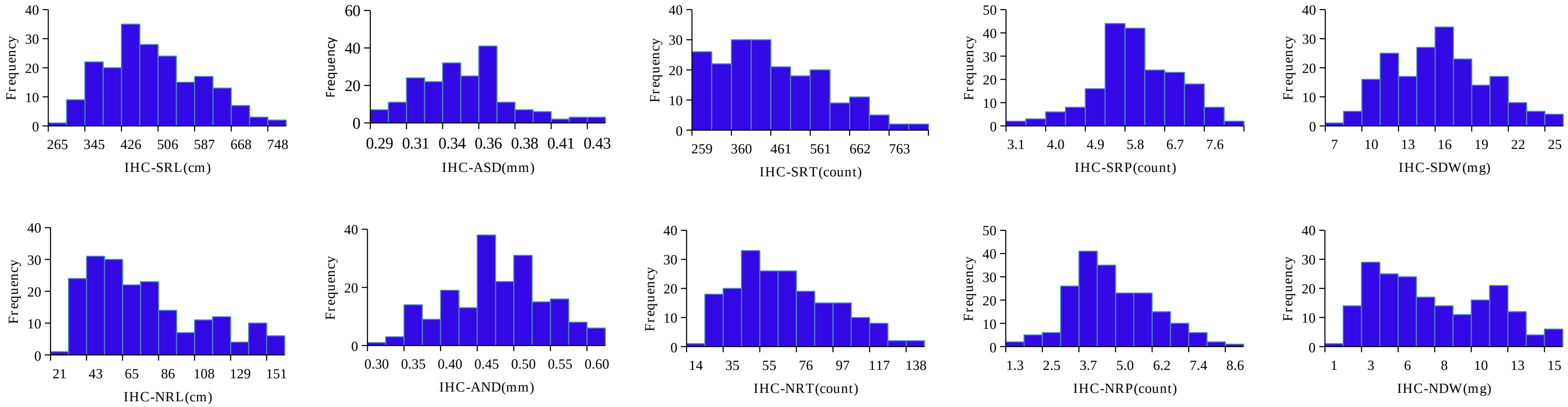

IHC-SRL(cm)
IHC-ASD(mm)
IHC-SRT(count)
IHC-SRP(count)
IHC-SDW(mg)
IHC-NRL(cm)
IHC-AND(mm)
IHC-NRT(count)
IHC-NRP(count)
IHC-NDW(mg)
Frequency

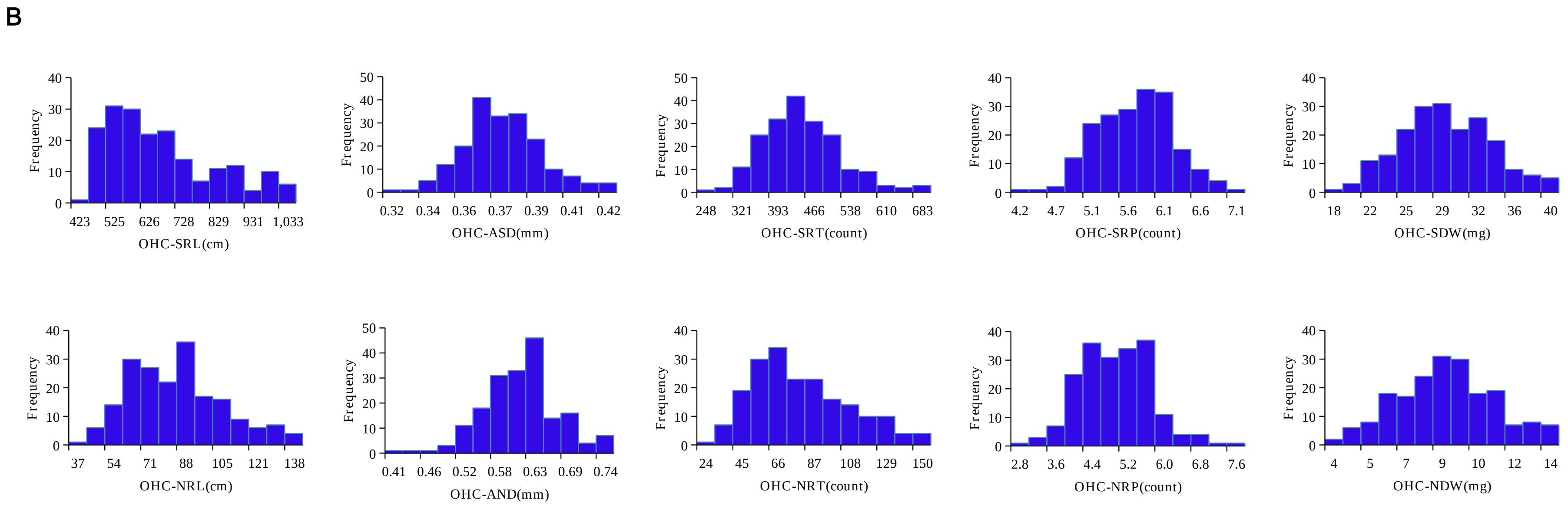
B
Frequency
OHC-SRL(cm)
423 525 626 728 829 931 1,033
OHC-ASD(mm)
0.32 0.34 0.36 0.37 0.39 0.41 0.42
OHC-SRT(count)
248 321 393 466 538 610 683
OHC-SRP(count)
4.2 4.7 5.1 5.6 6.1 6.6 7.1
OHC-SDW(mg)
18 22 25 29 32 36 40
OHC-NRL(cm)
37 54 71 88 105 121 138
OHC-AND(mm)
0.41 0.46 0.52 0.58 0.63 0.69 0.74
OHC-NRT(count)
24 45 66 87 108 129 150
OHC-NRP(count)
2.8 3.6 4.4 5.2 6.0 6.8 7.6
OHC-NDW(mg)
4 5 7 9 10 12 14

C

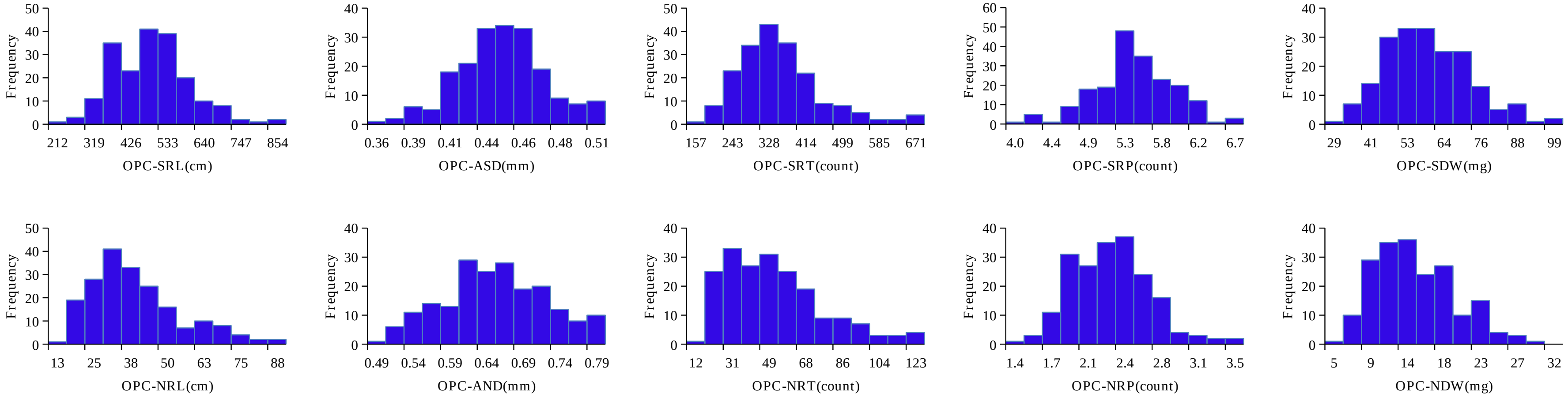

Figure S1 Plots of the frequency distributions of root traits under different growth environments. A, IHC; B, OHC; C, OPC
