# Supplementary figures and images for: Genome-Wide Association Study on Seminal and Nodal Roots of Wheat Under Different Growth Environments
Source: Front Plant Sci. 2021 Jan 11;11:602399. doi: 10.3389/fpls.2020.602399 (PMC7829178; doi:10.3389/fpls.2020.602399)

A

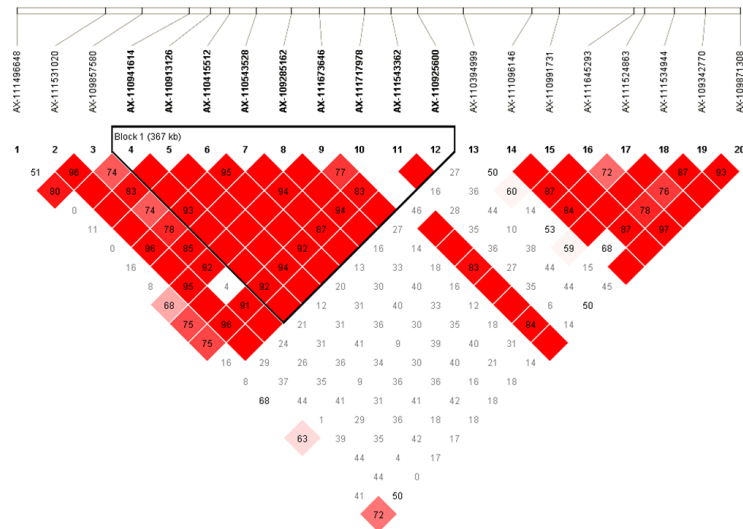

B

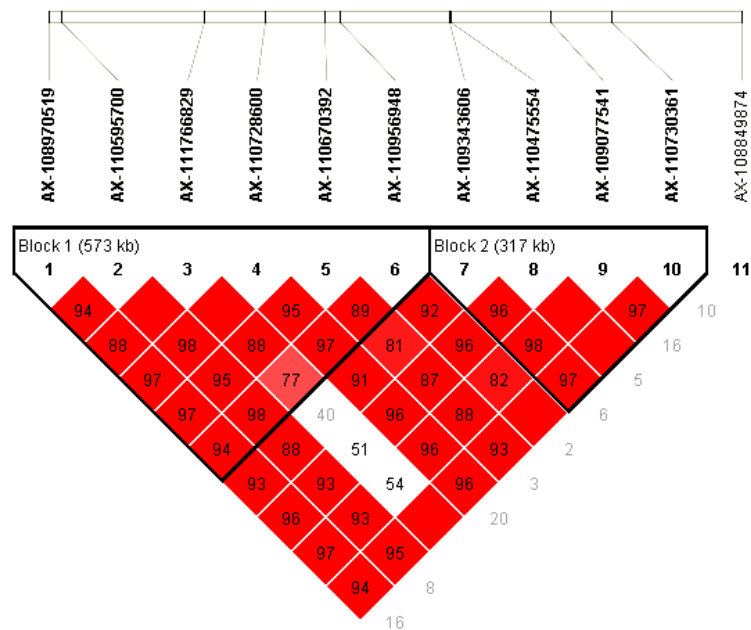

**Figure S2** Haploblock for local LD decay within the candidate region on chromosomes 2B and 7A

Supplement: Supplementary file 3 [file Data_Sheet_2.pdf]
